# Barriers to effective communication between family physicians and patients in walk-in centre setting in Dubai: a cross-sectional survey

**DOI:** 10.1186/s12913-018-3457-3

**Published:** 2018-08-14

**Authors:** Abdulaziz H. Albahri, Alya S. Abushibs, Noura S. Abushibs

**Affiliations:** Medical Education Department, Dubai Healthcare Authority, P.O. Box: 4545, Dubai, United Arab Emirates

**Keywords:** Communication barriers, Physician-patient relations, Family practice, Family physicians, Patient participation, Professional-patient relations

## Abstract

**Background:**

Effective communication between family physicians and their patients is crucial to improving healthcare outcomes and patients’ satisfaction. However, the barriers to effective communication have been weakly studied in the Gulf region with no reported studies in Dubai. This study aims to identify the main perceived barriers to effective communication between patients and their family physicians in Dubai from both the physicians’ and the patients’ viewpoints.

**Methods:**

The study was conducted at 12 primary healthcare centres in Dubai between October 2016 – July 2017. Two self-administered questionnaires were used, one measuring the patients’ perceived frequency of encounters with barriers to communication, while the other was for the family physicians’ perceived level of risk to communication posed by the barriers. The barriers were assessed in the following four domains: personal characteristics and attitudes, organisational factors, communication of information, and linguistic and cultural factors.

**Results:**

There were a total of 1122 patients and 170 family physicians, with 75% and 85% response rates, respectively. Having a time limitation was the highest ranking barrier, with 23.4% of patients encountering it half of the time-always, and 50.6% of physicians perceiving it as moderate-very high risk. This was followed by barriers in the communication of information domain, especially not checking the patient’s understanding and not educating the patient (16.0–16.9%) from the patients’ perception and presentation with multiple problems and not following with a treatment plan (51.2% and 35.9%, respectively), from the physicians’ perception. Preoccupation with medical records ranked in the second pentile for the physicians, and in the lowest pentile for the patients. Barriers related to the failure of rapport building and linguistic/cultural factors ranked in the fourth and fifth pentiles for both patients and physicians.

**Conclusion:**

Time pressure is the major perceived barrier to communication between patients and family physicians. In addition, a greater focus needs to be placed on training the physicians to convey their messages to the patients clearly, checking their understanding and managing poor historians.

**Electronic supplementary material:**

The online version of this article (10.1186/s12913-018-3457-3) contains supplementary material, which is available to authorized users.

## Background

The practice of good communication skills helps in building a trustful relationship between the physician and the patient, and enables a better understanding of the patient’s issues. This in turn results in improved diagnosis and the management of the patient’s condition [1]. With both sides being satisfied with the consultation and management, increased adherence to treatment and better quality of health care are desired outcomes [2].

There are elements that lead to effective communication between physicians and patients, and which can be attributed to factors associated with the physician, the patient, and the organisation or clinical set up [3]. The physician should strive to build a good rapport with the patient, and needs to address his/her concerns, as well as meeting expectations in an effective manner. The patient needs to be given the time and opportunity to fully express him or herself with regard to feelings, opinions, and information, in a setting where the patient feels his/her privacy is being respected. Only with this good shared understanding and shared management plan can the desired outcomes of effective communication be achieved [4].

Barriers to effective communication, on the other hand, fall into the same categories [3]. Time management, difficulties with rapport building, patients that are poor historians, physicians that are not explaining the condition and management effectively to their patients, the language and the culture of the patient and physician, and the physical set up of the clinic are all potential barriers that have been shown to affect communication between physicians and patients [1–3]. These factors have been studied in different parts of the world to various degrees; however, in the Arabian Peninsula, there is a limited number of studies that have been conducted to assess and address the subject [5–7]. In the United Arab Emirates (UAE), in particular, the subject is very weakly studied, with no major recent studies conducted in the field.

We conducted our study in the emirate of Dubai, which is one of seven emirates that constitute the UAE. It is a cosmopolitan city, with an estimated population of about two and a half million people, a 2.5% illiteracy rate and about 1% elderly population. The local UAE citizens make up only about 9% of the population, with the rest being from various parts of the world, mostly from South East Asian countries and various Arab countries [8]. With this diversity in the culture and population, and a lack of recent well-structured research on effective communication in the region, our study aims to identify the main perceived barriers to effective communication between patients and their family physicians in Dubai from both the physicians’ and the patients’ viewpoints.

## Methods

### Study setting

This study was conducted at all the primary healthcare centres of Dubai Healthcare Authority (DHA). DHA is the main governmental organisation that provides healthcare services to Dubai’s population. At the time of the study, which was from October 2016 until July 2017, there were 12 active primary healthcare centres. They function similar to walk-in centres, without a prior appointment system. The patient might choose to see the same family physician or another one based on availability or the patient’s personal preference. After registration, the patient sees a nurse, who makes an initial assessment and takes the patient’s vitals prior to the patient seeing the family physician. Each patient is allocated a 12 min consultation with the family physician. During the time of the study, the medical records were partially electronic, while the medical notes were on paper. Further, investigations and medications were both electronic. However, it is worth noting that from August 2017, i.e. after the study was completed, the primary healthcare centres at DHA went fully electronic by the use of the Epic® electronic medical record and the consultation time has been increased to 15 min.

### Study populations and sample size

The survey of patients had the following inclusion criteria: adult patients, aged 18 years of age and older, following a primary healthcare centre at DHA, and to be literate of either Arabic or English.

On the other hand, the physicians’ survey was exclusively performed on family physicians that were practising at DHA, as well as senior family medicine residents in their final year of training at DHA or who finished their training and were awaiting their professional promotion into specialist registrars.

The sample size was calculated using Epi Info™ v7.2 software by the Centres for Disease Control and Prevention (CDC). Based on a 95% confidence level and 3% margin of error, the needed patients’ sample size was calculated to be 1065 and the family physicians sample size was 168.

### Study design

This study was a cross-sectional survey based on two self-administered questionnaires: one designed for family physicians and the other designed for patients. The study participants were selected through convenience sampling. Each healthcare centre was provided with a set of patients’ questionnaires proportional to the population served, with a total of 1500 distributed questionnaires. Consecutive patients were provided with the questionnaire and asked to complete it prior to their entry to see their family physician. They had the choice of completing an Arabic or an English version of the questionnaire. The family physicians’ questionnaires, on the other hand, were provided to the head of each primary healthcare centre and they were asked to distribute them to their family physicians. A total of 200 questionnaires were distributed.

### The questionnaires

The questionnaires were mainly based on Part C of the Doctor-Patient Communication Needs Assessment Scale [9, 10]. Some adjustments were made to account for the local culture and setting. Few additional items were added from other sources, as described in Table 1. Samples of the final questionnaires used are provided in the Additional file 1 document. The patients’ questionnaire was translated into Arabic and piloted on a small group of patients to ensure consistency with the English version.

**Table 1:** List of the domains, subdomains, the barriers to physician-patient communication, and the literature sources used in the construction of the questionnaires

Domain	Subdomain	Patient survey		Source	Physician survey		Source
		Q #	Brief description	First author, year of publication, [reference no.]	Q #	Brief description	First author, year of publication, [reference no.]
Personal Characteristics and Attitudes	Failure of rapport building	6	Physician’s lack of interest in the issues raised	Lovell 2010 [9], Shapiro 2002 [10]	11	Difficulty establishing rapport	Lovell 2010 [9], Shapiro 2002 [10]
		10	Physician’s lack of empathy	Lovell 2010 [9], Shapiro 2002 [10]	16	Patient’s lack of trust with physician	Lovell 2010 [9], Shapiro 2002 [10]
		13	Unsatisfactory physician manners	Lovell 2010 [9], Shapiro 2002 [10]	13	Patient’s lack of interest in building a partnership with physician	Lovell 2010 [9], Shapiro 2002 [10]
Communication of Information	Lack of shared understanding pertaining to history and symptoms	11	Not addressing all the issues raised	Lovell 2010 [9], Shapiro 2002 [10]	6	Presentation with multiple problems	Lovell 2010 [9], Shapiro 2002 [10]
		12	Difficulty understanding the problem	Lovell 2010 [9], Shapiro 2002 [10]	7	Disorganised history by patient	Lovell 2010 [9], Shapiro 2002 [10]
					9	Inconsistent information provided by patient	Lovell 2010 [9], Shapiro 2002 [10]
	Lack of shared understanding pertaining to diagnosis				3	Difficulty getting the patient to understand the diagnosis	Lovell 2010 [9], Shapiro 2002 [10]
					4	Patient’s difficulty in understanding implications of diagnosis	Lovell 2010 [9], Shapiro 2002 [10]
					12	Difficulty reconciling patient self-diagnosis with physician’s diagnosis	Lovell 2010 [9], Shapiro 2002 [10]
	Lack of shared decision-making and management	14	Pressurising patient on decision-making	Lovell 2010 [9], Shapiro 2002 [10]	2	Patient not following through with treatment plan	Lovell 2010 [9], Shapiro 2002 [10]
		15	Lack of aid tools	Turner 2009 [25]	8	Patient not buying into treatment plan	Lovell 2010 [9], Shapiro 2002 [10]
					10	Patient’s lack of interest in self-care	Lovell 2010 [9], Shapiro 2002 [10]
	Lack of communication attributes	2	Amount of information provided by physician- large	Lovell 2010 [9], Shapiro 2002 [10]			
		7	Speech rapidity	Lovell 2010 [9], Shapiro 2002 [10]			
		8	Denying patient the opportunity to ask questions and talk	Lovell 2010 [9], Shapiro 2002 [10]			
		9	Not confirming understanding	Lovell 2010 [9], Shapiro 2002 [10]			
Linguistic and Cultural Factors	Use of medical jargon	3	Use of medical jargon	Lovell 2010 [9], Shapiro 2002 [10]			
	Lack of cultural competency	16	Difficulty understanding culture/health beliefs	Lovell 2010 [9], Shapiro 2002 [10]	15	Cultural beliefs interference with diagnosis and management	Lovell 2010 [9], Shapiro 2002 [10]
		18	Physician’s culture/nationality	Roter 2006 [3]	17	Unfamiliarity with cultural alternative therapies	Lovell 2010 [9], Shapiro 2002 [10]
	Difficulty with language use	4	Difficulty with language/dialect	Mira 2012 [26], Shapiro 2002 [10]	18	Difficulty with dialect/language	Mira 2012 [26], Shapiro 2002 [10]
					5	Inadequate translation by interpreter	Lovell 2010 [9], Shapiro 2002 [10]
					14	Inappropriate interpreter	Lovell 2010 [9], Shapiro 2002 [10]
Organisational Factors	Time limitation	1	Consultation time limitation	Lovell 2010 [9], Shapiro 2002 [10]	1	Consultation time limitation	Lovell 2010 [9], Shapiro 2002 [10]
	Medical records use	5	Preoccupation with computer/mobile phone	Shachak 2009 [27]	19	Preoccupation with medical records	Shachak 2009 [27]
	Physical set up				20	Unsuitability of the physical set up	Ajiboye 2015 [28]
	Physician’s gender	17	Physician’s gender	Roter 2006 [3]			

Q # denotes the number of the barrier as it was asked in Section II of the corresponding questionnaire

For analysis purposes, the barriers to communication were grouped into four major domains, as described in Table 1: personal characteristics and attitudes, communication of information, linguistic and cultural factors, and organisational factors [11]. The barriers were confined to the consultation room; thus, barriers associated with or affected by other external factors were not explored.

For the patients’ perceived encounter with the barriers to communication, patients were asked to mark each barrier in terms of frequency at one of five levels from never to always.

**Fig. 1:**
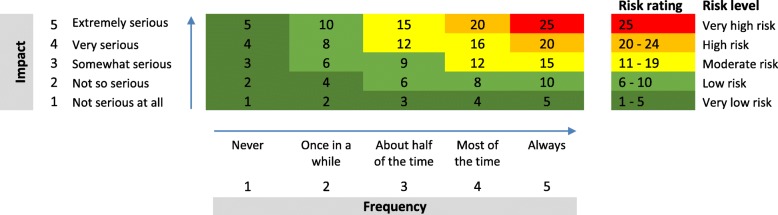
The frequency-impact matrix for calculating the family physicians’ perceived level of risk posed by the barriers to communication with their patients. The numbers in the colour-coded matrix are the results of multiplying the score of the frequency by the score of the impact (seriousness)

The physicians, on the other hand, were asked to mark each barrier from two perspectives: frequency and seriousness, each marked at five levels (never to always for frequency, and not at all serious to extremely serious for the latter). The lowest of each choice was given one point, while the highest was given 5 points. A perceived qualitative level of risk stratification was generated by multiplying the perceived frequency of the barrier by the perceived seriousness of the barrier [12]. By level of risk we mean the degree at which a barrier to communication might adversely affect the outcome of the communication. This has two dimensions; a perceived frequency of encounter with the barrier, and a perceived seriousness or impact that the barrier might have on the communication once it has been encountered. There are a total of 25 possible scores, ranging from 1 to 25 and leading to five possible qualitative levels of risk, ranging from very low to very high risk groups, as summarised in the frequency-impact matrix (Fig. 1) [12].

### Statistical analysis

Data were entered into Excel® sheet, and further analysis was performed using GraphPad Prism® v7 statistical analysis software. Pearson’s chi square test of independence was used for categorical nominal data analysis of association, and Fisher’s exact test with two-tailed *p*-value was applied in the analysis of 2*2 contingency Tables. *P* value < 0.05 was considered to be statistically significant.

### Ethical considerations

This study was approved by the Research Ethics Committee at DHA. Participation in the study was voluntary and participants were provided with an information sheet about the study. Moreover, written consents were obtained.

## Results

### Patients’ socio-demographic data and general satisfaction

There were a total of 1122 patients participating in the study (75% response rate). Socio-demographic and patient satisfaction data are summarised in Table 2. The majority were female (68.3%) and mostly from relatively young age groups, with patients aged above 50 years constituting only 9.8% of the participants. Of whom, only 20 patients were above 65 years old. Most of the patients were UAE nationals (77.2%), while the other patients were from different countries, with at least 8.3% coming from non-Arab countries. Nearly 86% of the participants stated their first language was Arabic, while English was the first language of about 6% of the patients. The patients had good educational backgrounds, with approximately 94% of them having at least a high school education.

**Table 2:** Patients’ socio-demographic characteristics and satisfaction data (*n* = 1122)

Category	Subcategory	Count	%
Age (in years)	18–30	461	41.1%
	31–40	335	29.9%
	41–50	216	19.3%
	51–65	90	8.0%
	> 65	20	1.8%
Gender	Male	356	31.7%
	Female	766	68.3%
Nationality	United Arab Emirates	866	77.2%
	Other	256	22.8%
	Arab	54	4.8%
	Non-Arab	93	8.3%
	Unspecified	109	9.7%
First language	Arabic	964	85.9%
	English	66	5.9%
	Other	92	8.2%
Educational level	None, or partial	48	4.3%
	Vocational/Technical	23	2.1%
	High school	460	41.0%
	Undergraduate	446	39.8%
	Postgraduate	145	12.9%
Number of visits within last year	0	76	6.8%
	1	91	8.1%
	2–5	506	45.1%
	6–10	228	20.3%
	> 10	221	19.7%
Purpose of current or most recent visit	New symptoms	213	19.0%
	Follow up	549	48.9%
	Repeat prescription	183	16.3%
	Other	177	15.8%
Perceived general health status	Poor	86	7.7%
	Normal	831	74.1%
	Very good	205	18.3%
Satisfaction with family physician	Not satisfied	22	2.0%
	Average satisfaction	578	51.5%
	Very satisfied	522	46.5%

About 45% of patients claimed to have 2–5 visits to their family physician within the last year, and 40% had at least 6 visits. Follow up and need for repeat prescriptions were the most stated reasons for the current or most recent visit (65.2%). Most patients viewed their general health as normal or very good (92.4%) and were satisfied with their family physicians (only 2% unsatisfied).

### Patients’ perceived frequency of encounter with the barriers to communication

The patients’ perceived frequencies of encounters with barriers to communication were ranked based on the half of the time- always subgroup in descending order of pentiles, as summarised in Table 3. The frequencies ranged from 23.4% for the highest rank to 11.1% for the lowest rank. Having limited time during the consultation was the only barrier in the first pentile, with 23.4% frequency. There were no barriers in the second pentile. Other elements of the organisational factors domain, including physician’s gender and medical record use, were in the fourth and the fifth pentiles, with 14.3% and 12.5% frequencies, respectively. There were no statistically significant associations between the physician’s gender as a barrier and the patient’s gender or nationality (Additional file 2: Table S1).

**Table 3:** Patients’ perceived frequency of encounter to the barriers of communication ranked in descending order of pentiles and grouped by domains (*n* = 1122)

Domain	Subdomain	Pentile ranking	Barriers to communication	Frequency of encounter			
				Half of the time- Always		Once in a while- Never	
				Frequency	Percent	Frequency	Percent
Organisational Factors	Time limitation	1	Having limited time during the consultation	263	23.4%	859	76.6%
	Physician’s gender	4	Not having a physician of the same gender	160	14.3%	962	85.7%
	Medical record use	5	The physician is preoccupied with computer/phone	140	12.5%	982	87.5%
Communication of Information	Lack of communication attributes	3	The physician providing large amounts of information	190	16.9%	932	83.1%
	Lack of shared management	3	Not having aid tools, like brochures/writing materials	190	16.9%	932	83.1%
	Lack of communication attributes	3	The physician is not checking the patient’s understanding	180	16.0%	942	84.0%
	Lack of shared understanding of history	4	The physician is not addressing all the issues raised by the patient	170	15.2%	952	84.9%
	Lack of shared understanding of history	4	Inability of the physician to understand the patient’s problem	166	14.8%	956	85.2%
	Lack of communication attributes	4	The physician is talking too fast	156	13.9%	966	86.1%
	Lack of communication attributes	4	The physician is not giving the patient a chance to talk/ask questions	153	13.6%	969	86.4%
	Lack of shared management	5	The physician is pressurising the patient into making quick decisions	126	11.2%	996	88.8%
Personal Characteristics and Attitudes	Failure of rapport building	4	The physician is not showing interest in the patient’s issues	171	15.2%	951	84.8%
	Failure of rapport building	4	The physician is not being empathic	169	15.1%	953	84.9%
	Failure of rapport building	5	The physician’s manners unsatisfactory	124	11.1%	998	89.0%
Linguistic and Cultural Factors	Lack of cultural competency	4	Not having a physician of the same nationality/culture	177	15.8%	945	84.2%
	Medical jargon use	5	The physician is using medical jargon	138	12.3%	984	87.7%
	Difficulty with language use	5	Difficulty understanding the physician’s language/dialect	135	12.0%	987	88.0%
	Lack of cultural competency	5	The physician not understanding the culture/health beliefs of the patient	130	11.6%	992	88.4%

Elements of the communication of information domain were the only barriers in the third pentile, which included the physician providing a large amount of information, not having educational materials, and not checking the patient’s understanding. These were followed by elements of the lack of shared understanding pertaining to history subdomain, including not addressing all the issues raised by patients, as well as the physician talking rapidly and not giving the patient a chance to ask questions (frequencies ranged between 13.6–15.2%). However, placing pressure on patients to make decisions was one of the least ranked, with only 11.2% of patients perceiving it as half of the time- always frequency (fifth pentile).

Barriers related to failure of rapport building were in the fourth pentile (approximately15%), except for unsatisfactory manners of the physician, which ranked the least compared to all other barriers of communication (11.1%). The linguistic and cultural factors domain was one of the least ranked as well, with most elements being in the fifth pentile, including the use of medical jargon and difficulty understanding the physician’s language or the patient’s culture/health beliefs. The only exception was not having a physician of the same culture, which was in the fourth pentile (about 15.8%). There were no statistically significant associations between either subdomain of lack of cultural competency or difficulty with language use and the patient’s nationality or first language (Additional file 2: Table S1).

### Family physicians’ socio-demographic and satisfaction data

There were a total of 170 family physicians participating in the study (85% response rate). Table 4 summarises the socio-demographic data. The participants were mostly in the third and fourth decades of their life (41.8% and 25.3%, respectively). Female physicians constituted the majority, with nearly 78% of all participants. There were more non-UAE national physicians (55.9%), of whom at least 20 physicians were from non-Arab countries (30 participants did not state their country of origin). Only about one fifth of the participants stated their first language being a language other than Arabic.

**Table 4:** Family physicians’ socio-demographic characteristics and satisfaction data (*n* = 170)

Category	Subcategory	Count	(%)
Age (in years)	≤30	25	14.7%
	31–40	71	41.8%
	41–50	43	25.3%
	≥51	31	18.2%
Gender	Male	38	22.4%
	Female	132	77.7%
Nationality	United Arab Emirates	75	44.1%
	Other	95	55.9%
	Arab	45	26.5%
	Non-Arab	20	11.8%
	Unspecified	30	17.7%
First language	Arabic	138	81.2%
	Other	32	18.8%
	English	6	3.5%
	Farsi	1	0.6%
	Hindi	6	3.5%
	Urdu	17	10.0%
	Malayalam	2	1.2%
Professional level	Senior resident	25	14.7%
	Specialist registrar	72	42.4%
	Senior specialist registrar	60	35.3%
	Consultant	13	7.7%
Years of experience	≤5	21	12.4%
	6–10	37	21.8%
	11–15	32	18.8%
	16–20	38	22.4%
	> 20	42	24.7%
Communication skills training	Medical school		
	Yes	105	61.8%
	No	65	38.2%
	Postgraduate/Residency		
	Yes	151	88.8%
	No	19	11.2%
	Within last year		
	Yes	61	35.9%
	No	109	64.1%
Job satisfaction	Not satisfied	1	0.6%
	Average satisfaction	80	47.1%
	Very satisfied	89	52.4%
Communication skills satisfaction	Not satisfied	0	0.0%
	Average satisfaction	50	29.4%
	Very satisfied	120	70.6%

Most of the physicians were registrars or senior registrars (a total of 132), with only 13 consultants and 25 senior residents. Eighty of the participants had more than 15 years of experience, constituting almost half of the physicians (47.1%). The postgraduate/residency period was the highest period that the participants had received communication skills training (88.8%); while during medical school, only 61.8% stated that they received formal training, and more than half (64.1%) stated that they did not receive formal training over the past year. Overall, all the family physicians were generally satisfied or were very satisfied with their jobs, as well as with their communication skills (there was a single physician who expressed dissatisfaction with the job).

### Family physicians’ perceived level of risk posed by the barriers to communication

The barriers to communication from the physicians’ perceptions were stratified into five levels, ranging from very low to very high, based on the level of risk calculation, as was mentioned in the methodology section. They were further grouped into a very low-low level of risk group and moderate-very high (M-VH) group. As shown in Table 5, the barriers were ranked in descending order of pentiles, as per the M-VH level of risk posed by the barrier.

**Table 5:** Family physicians’ perceived level of risk posed by the barriers to effective communication, ranked in descending order of pentiles and grouped by domains (*n* = 170)

Domain	Subdomain	Pentile ranking	Barriers to communication	Level of risk			
				Moderate- Very high		Very low- Low	
				Frequency	Percent	Frequency	Percent
Organisational Factors	Time limitation	1	Consultation time limitation	86	50.6%	84	49.4%
	Medical record use	2	Preoccupation with medical records	59	34.7%	111	65.3%
	Physical set up	5	Unsuitability of the physical set up	10	5.9%	160	94.1%
Communication of Information	Lack of shared understanding of history	1	Presentation with multiple problems	87	51.2%	83	48.8%
	Lack of shared management	2	Patient not following through with treatment plan	61	35.9%	109	64.1%
	Lack of shared understanding of history	3	Disorganised history by patient	49	28.8%	121	71.2%
	Lack of shared management	4	Patient’s lack of interest in self-care	30	17.7%	140	82.4%
	Lack of shared understanding of history	4	Inconsistent information provided by patient	29	17.1%	141	82.9%
	Lack of shared management	4	Patient not buying into treatment plan	26	15.3%	144	84.7%
	Lack of shared understanding of diagnosis	4	Patient’s difficulty understanding implications of diagnosis	23	13.5%	147	86.5%
	Lack of shared understanding of diagnosis	5	Difficulty reconciling patient self-diagnosis with physician’s diagnosis	13	7.7%	157	92.4%
	Lack of shared understanding of diagnosis	5	Difficulty getting patient to understand the diagnosis	8	4.7%	162	95.3%
Linguistic and Cultural Factors	Difficulty with language use	4	Inadequate translation by interpreter	26	15.3%	144	84.7%
	Lack of cultural competency	4	Cultural beliefs interference with diagnosis and management	26	15.3%	144	84.7%
	Lack of cultural competency	5	Unfamiliarity with cultural alternative therapies	21	12.4%	149	87.7%
	Difficulty with language use	5	Difficulty with dialect/language	10	5.9%	160	94.1%
	Difficulty with language use	5	Inappropriate interpreter	9	5.3%	161	94.7%
Personal Characteristics and Attitudes	Failure of rapport building	5	Patient’s lack of interest in building a partnership with physician	11	6.5%	159	93.5%
	Failure of rapport building	5	Patient’s lack of trust with physician	8	4.7%	162	95.3%
	Failure of rapport building	5	Difficulty establishing rapport	7	4.1%	163	95.9%

Patients presenting multiple problems and consultation time constraints were ranked the highest, with almost half of all physicians perceiving them in the M-VH level of risk (about 51% in each barrier group). There were no statistically significant associations between either of these two barriers and the level of seniority of the physician or the communication skills training received in the past (Additional file 2: Table S2). When looking at other barriers in the organisational factors domain, preoccupation with medical records ranked high, as well being in the second pentile, with about one third of physicians perceiving it as M-VH risk. On the other hand, the physical set up of the consultation room ranked low in the fifth pentile.

In addition to the presentations with multiple problems barrier, other elements of the communication of information domain ranked generally higher compared to other domains. Patients not following through with treatment plans, and ones that had a disorganised history ranked in the second (35.9%) the third (28.8%) pentiles, respectively. Patients that lacked interest in self-care, that did not buy into the treatment plan, and that provided inconsistent information, all ranked in the fourth pentile. All elements related to the subdomain of lack of shared understanding pertaining to diagnosis ranked low in the fourth and fifth pentiles, ranging from about 4.7–13.5% of physicians perceiving them as M-VH risk to communication.

Elements of the linguistic and cultural factors domain generally ranked in the lowest two pentiles, with only 5.3–15.3% of the family physicians considering them as M-VH risk. Inadequate translation by interpreters was one of the highest ranked in this domain. There were statistically significant associations between this barrier and both the nationality of the physician and their primary language (both *p*-values < 0.05). More UAE national physicians and Arabic speaking physicians considered this barrier as M-VH risk.

Lastly, all barriers related to failure of rapport building ranked in the fifth pentile, with only 4.1–6.5% of physicians viewing them as M-VH risk to the communication.

## Discussion

Our study highlights multiple similarities and discrepancies between patients’ views and family physicians’ views of the encounters with the barriers to communication and the level of risk posed by them. First, time limitation was ranked in the top pentile by patients and was also viewed in the top pentile by physicians as a risk to communication. This echoes multiple studies conducted worldwide and highlights the importance of time management and the effect of time limitations on the consultation [13, 14]. In our study setting, where patients approached their family physicians without prior appointments, i.e. similar to a walk-in centre, this effect might be more exaggerated, as the attending physician might be taken by surprise in regard to the number of patients turning up in a particular day or by an emergency case that would limit the time available to the other awaiting patients.

Additionally, physicians viewed patients that presented with multiple problems, who were poor historians, and the ones not engaging and adhering to the treatment plan, as major barriers to communication. Patients, on the other hand, viewed physicians that were not educating them or checking their understanding as higher frequency encounter barriers. Similar findings pertaining to patients’ views were found in a study conducted by Harrison in another city of the UAE (Al-Ain) in 1996, which showed patients giving higher ratings to physicians who discussed and explained the condition and management to the patients [5]. In a study by Lovell and colleagues, the aforementioned barriers also ranked higher by physicians; however, in their study, barriers pertaining to diagnosis and its explanation ranked higher compared to our study, where they ranked in the lowest pentile [9]. This could be due to over confidence from the physicians’ side in regards to their ability to explain the diagnoses and their implications to their patients.

When looking at medical record use, physicians perceived it as one of the highest barriers to pose significant risk to their communication with patients. The patients, on the other hand, viewed the physician’s preoccupation with medical records in the lowest pentile as a barrier to communication. This mirrored a recent study conducted by Shaarani and colleagues in Lebanon, which found that two-thirds of patients did not consider the physician’s preoccupation with medical records negatively impacting their communication [15]. Consequently, this issue might represent an element of personal stress on the side of the physician rather than being of great hindrance to communication. It will be of great interest to see if the physicians’ view changes after the implementation of a fully electronic medical record compared to the partial electronic record that was in place during the time of the study.

Elements related to failure of rapport building in general ranked as the least in terms of risk posed to communication, with only 7–11 family physicians perceiving any of the elements as M-VH risk. On the other hand, there were 11.1–15.2% of patients who claimed that they face issues pertaining to rapport building half of the time-always. We speculate that this discrepancy is due to the physicians overestimating their rapport building skills, or not paying great attention to it and thus, assuming good knowledge and practice without real application [1, 16]. In view of the time pressure being ranked in the first pentile, some authors suggest that the response to time pressures is either limited rapport building during a good consultation, or no rapport building during the low quality consultations [17]. Further studies would be needed to delineate which of these two groups the majority of the physicians in our study fall into.

Despite the multi-cultural setting of the healthcare system in Dubai, both patients and physicians viewed barriers related to language and culture in the lowest pentiles. This could be explained by the multicultural background of the family physicians, as more than half of them were non-UAE nationals. There have been multiple studies showing that patients of different cultural groups being attended by physicians of the same culture or by ones who speak the language had better ratings for physicians and better communication outcomes [18–20].

While Dubai’s population is predominantly male (72.2%) mainly due to the expatriate male work force, the patients in our study were mostly females (68.3%) [8]. There are two main explanations we can provide for this discrepancy: the first is that the expatriate patients tend to visit private healthcare centres due to the cost and the insurance coverage, and thus their number at the governmental healthcare centres is less. The second explanation is that females are more likely to seek medical attention compared to males as suggested by various studies [3]. However, we did not find a statistically significant association between being able to see a physician of the same gender and the patient’s gender, despite the fact that we were speculating that in a somewhat conservative culture we would find more female patients stating that not having a physician of the same gender is a hindrance to the communication. We believe that this is mostly due to the fact that the family physicians in our study setting are predominantly females (77.7%). On the other hand, there was a statistically significant higher percentage of the female patients compared to the percentage of the male patients who noted that there was time limitation during the consultation, that not all their issues have been addressed, that the physician did not give them the chance to talk, nor that their problem was fully understood by the physician (Additional file 2: Table S4). This echoes various studies highlighting that female patients tend to ask more questions and engage in more reciprocal discussion with the physicians [3]. Therefore, in our study setting where family physicians feel pressurised for time, we believe that female patients are more likely to feel the impact of this pressure since they do not get enough time to discuss their issues and ask questions. Further studies would need to be conducted to look into the structure of the consultation and how it is impacted by the gender of the patient and the physician in the current setting.

It is worth noting that only 2% of the patients were unsatisfied with their family physicians despite all the above mentioned barriers. Additionally, none of the family physicians were unsatisfied with their communication skills; rather, there were more physicians who were very satisfied compared to the average satisfaction group. Once again, this might highlight the issue of overestimation of one’s own communication skills abilities on the side of physicians.

In our study, we failed to find any statistically significant associations between the previous training in communication skills at any of the main stages of the physician’s career, and either of the highest two perceived barriers by family physicians, mainly time limitations and patients presenting multiple problems. While our study had broad questions in regard to receiving training in communication skills, it is worth highlighting that the type of training and its duration might all play a greater role in terms of outcome. There are multiple studies in the literature that showed different outcomes about training; generally speaking, short, brief workshops were less effective compared to more intense longer training periods [21–24].

### Study limitations

There are multiple limitations to our study. Firstly, the patient population is primarily young adult, and the elderly, especially the over 65 years old, constituted a smaller percentage of the patients. Therefore, the results mostly reflect the views of the younger age groups. Nonetheless, the over 50 years old make 6–7% of Dubai’s population, with the over 65 years old making less than 1.5% of the population [8]. This is not very far from the population in our study where we had 9.8% of the patients over the age of 50 years. However, with only 20 patients being over the age of 65, there is limited data that can be extrapolated with statistical significance. Nevertheless, when looking at the entire group i.e. all patients aged above 50 years as compared to the younger age groups, there were statistically significant associations with all the barriers to communication and the patients’ age groups (with the exception of the physician’s gender barrier. Additional file 2: Table S5). In general, there were fewer percentages of the older patients perceiving the encounter with the barriers to effective communication as half of the time- always compared to the younger age groups. This highlights the evidence shown in some previous studies that physicians tend to give the older patients more time, explain more, and clarify ambiguities when dealing with older compared to younger patients [3]. However, we believe our study was not adequately designed to look into the communication challenges facing the geriatric population as they are more complex, tend to present with multiple chronic diseases, be accompanied by another person such as a relative that might affect the communication, and might need to be dealt with in a different setting such as their own homes or nursing homes [3]. Therefore, a study designed to look specifically at the geriatric population would provide greater insight into the barriers faced by this age group.

Furthermore, the questionnaire was provided in Arabic and English; consequently, illiterate patients and the ones who do not speak these languages were not included in the study. Lastly, due to the unique setting the study was conducted in, i.e. family physicians working in a walk-in centre setting, where patients might opt to see the same family physician or a different physician at each visit; the views of the patients were either reflecting their opinion about a single family physician or their general impression about multiple physicians. However, we believe these views provide a realistic representation of how the patients generally perceive communication barriers in real life. While some patients pointed out in the comments section of the questionnaire (data not shown) that they prefer to be followed up by a single physician, this area needs to be further researched at both the patients and family physicians level to delineate the challenges and the best way to implement such a system, as well as to assess its effect on patient-physician communication.

## Conclusions

This study sheds light on the barriers faced by family physicians working in a walk-in centre like environment, with both patients and physicians being from multicultural, predominantly Arabic-speaking backgrounds. Time limitations are perceived as the biggest hindrance to communication. Both patients and family physicians views highlight the importance of effective explanations of the medical condition and its management, and the need from the physicians’ side for greater focus on managing patients who are poor historians or who are showing a lack of interest in adhering to the management plan. Family physicians seemed to be over confident with their rapport building skills when compared to patients’ views. With both physicians and patients being from multicultural backgrounds, linguistic and cultural factors in general did not rank high in either list of the barriers. Further studies are needed to delineate the causes and solutions of time pressure, including a comparison of the newly implemented and fully electronic medical records, as compared to the partial electronic records that were in place during the time of the study. The best measures to improve physicians’ abilities to deal with challenging patients and enhance their skills to educate their patients would need to be tested and investigated as well.

## Additional files

Additional file 1: Patients’ questionnaire sample. Family physicians’ questionnaire sample. (PDF 213 kb)

Additional file 2: **Table S1**. Associations between patients’ gender, nationality, and first language against potential related barriers to communication in the linguistic and cultural factors domain, as well as physicians’ gender (*n* = 1122). **Table S2.** Associations between family physicians’ professional level, as well as communication skills training against the time limitation and multiple problem presentation barriers of communication (*n* = 170). **Table S3.** Associations between family physicians’ nationality, as well as first language and the inadequate translation barrier to communication (*n* = 170). **Table S4.** Associations between patients’ gender and the barriers to communication with family physicians (*n* = 1122). **Table S5.** Associations between patients’ age and the barriers to communication with family physicians (*n* = 1122). (DOC 215 kb)

## Abbreviations

CDC: Centres for Disease Control and Prevention
DHA: Dubai Healthcare Authority
M-VH: Moderate-Very High
UAE: United Arab Emirates
