# Supplementary material for: Barriers to effective communication between family physicians and patients in walk-in centre setting in Dubai: a cross-sectional survey
Source: BMC Health Serv Res. 2018 Aug 14;18:637. doi: 10.1186/s12913-018-3457-3 (PMC6092839; doi:10.1186/s12913-018-3457-3)
Supplement: Supplementary file 1 — Patients’ questionnaire sample. Family physicians’ questionnaire sample. (PDF 213 kb) [file 12913_2018_3457_MOESM1_ESM.pdf]

GOVERNMENT OF DUBAI

هيئة الصحة بدبي
DUBAI HEALTH AUTHORITY

S.N. P

Patient Questionnaire

"Barriers to Effective Communication between Family Physicians and Patients and the Associated Factors at Dubai Healthcare Authority Primary Healthcare Centres—a Cross-Sectional Survey, 2016-2017"

Section I: Demographic data

Please provide the following information about yourself:-

1. Age: ☐ 18-30 ☐ 31-40 ☐ 41-50 ☐ 51-65 ☐ > 65

2. Gender: ☐ Male ☐ Female

3. Nationality: ☐ UAE ☐ Other country; please specify:

4. Your first language: ☐ Arabic ☐ English ☐ Farsi ☐ Urdu ☐ Hindi

☐ Other; please specify:

5. Spoken languages other than your first language (specify as many as applies):

☐ Arabic ☐ English ☐ Farsi ☐ Urdu ☐ Hindi

☐ Other; please specify:

6. Highest level of education completed: ☐ High school graduate ☐ Undergraduate ☐ Postgraduate studies

☐ Vocational/ Technical training ☐ No studies, or partial school studies

7. How many times did you visit your family doctor within the last year?

☐ 1 ☐ 2-5 ☐ 6-10 ☐ >10 ☐ Did not see him/her in the last year

8. What was the purpose of your last/current visit to your family doctor?

☐ New acute symptoms ☐ Follow up ☐ For prescription ☐ Other

9. How would you consider your own general health:

☐ Poor ☐ Normal ☐ Very good

10. How satisfied are you with your family doctor:

☐ Not satisfied ☐ Average satisfaction ☐ Very satisfied

11. Do you feel free to voice your opinion and express yourself during the consultation?

☐ Always ☐ Most of the time ☐ About half the time ☐ Once in a while ☐ Never

GOVERNMENT OF DUBAI

هيئة الصحة بدبي
DUBAI HEALTH AUTHORITY

Section II

The following are barriers that will hinder effective communication between patients and their doctors; for each statement please circle how frequently it has been a problem for you to effectively communicate with your family doctor

	Always a problem	Most of the time	About half the time	Once in a while	Never a problem
**1. Having limited time during the consultation**	5	4	3	2	1
**2. The doctor providing a large amount of information that is difficult to remember**	5	4	3	2	1
**3. The doctor using medical/scientific terms that I do not understand and s/he does not explain**	5	4	3	2	1
**4. The doctor speaking a language/dialect that I find difficult to understand**	5	4	3	2	1
**5. The doctor being more preoccupied by the computer/mobile phone than talking to me**	5	4	3	2	1
**6. The doctor not showing any interest in the issues I raise during the consultation**	5	4	3	2	1
**7. The doctor talking very fast**	5	4	3	2	1
**8. The doctor not pausing to give me a chance to talk or ask questions**	5	4	3	2	1
**9. The doctor not checking my understanding of what s/he has said**	5	4	3	2	1
**10. The doctor not being sympathetic to my concerns**	5	4	3	2	1
**11. The doctor not addressing all the issues I raise during the consultation**	5	4	3	2	1
**12. The doctor not being able to understand my problem**	5	4	3	2	1
**13. The doctor's manner is not satisfactory and he often talks down to me**	5	4	3	2	1
**14. The doctor putting pressure on me to make difficult decisions and not giving me time to think and decide**	5	4	3	2	1
**15. Not having aid tools such as leaflets/brochures, or pen and paper that I can write notes**	5	4	3	2	1
**16. The doctor not understanding my culture and/or health believes**	5	4	3	2	1
**17. Not having a doctor of the same gender**	5	4	3	2	1
**18. Not having a doctor of the same nationality/culture**	5	4	3	2	1

19. If you believe there are other barriers that hinder your communication with your family doctor and they are not stated above, please list them in the space below:

GOVERNMENT OF DUBAI

هيئة الصحة بدبي
DUBAI HEALTH AUTHORITY

S.N. D

Doctor Questionnaire

"Barriers to Effective Communication between Family Physicians and Patients and the Associated Factors at Dubai Healthcare Authority Primary Healthcare Centres—a Cross-Sectional Survey, 2016-2017"

Section I: Demographic data

Please provide the following information about yourself:-

**1. Age:**	☐ 25-30 ☐ 31-40 ☐ 41-50 ☐ 51-65
**2. Gender:**	☐ Male ☐ Female
**3. Nationality:**	☐ UAE ☐ Other country; please specify:
**4. Your first language:**	☐ Arabic ☐ English ☐ Farsi ☐ Urdu ☐ Hindi<br>☐ Other; please specify:

5. Spoken languages other than your first language (specify as many as applies):

☐ Arabic ☐ English ☐ Farsi ☐ Urdu ☐ Hindi

☐ Other; please specify:

6. Your professional level

☐ Senior resident ☐ Specialist registrar ☐ Senior specialist registrar ☐ Consultant

7. Years of experience since graduating from medical school

☐ 3-5 ☐ 6-10 ☐ 11-15 ☐ 16-20 ☐ >20

8. Have you received any structured formal training in communication skills during the following:-

- **Medical school** ☐ Yes ☐ No
- **Postgraduate level/Residency training** ☐ Yes ☐ No
- **Within the last year** ☐ Yes ☐ No

9. How satisfied are you with your job:

☐ Not satisfied ☐ Average satisfaction ☐ Very satisfied

10. How satisfied are you with your communication skills:

☐ Not satisfied ☐ Average satisfaction ☐ Very satisfied

Section II

The following are barriers that will hinder effective communication between patients and their doctors; for each statement please state how frequently you encounter the problem and how big of a problem it is once it has occurred.

	Frequency					Seriousness				
	Always	Most of the time	About half the time	Once in a while	Never	Extremely serious	Very serious	Somewhat serious	Not so serious	None
1. There is limited time during the consultation	5	4	3	2	1	5	4	3	2	1
2. The patient does not follow through with treatment or make lifestyle changes	5	4	3	2	1	5	4	3	2	1
3. Difficulty getting the patient to understand the diagnosis	5	4	3	2	1	5	4	3	2	1
4. Difficulty getting the patient to understand the implications of the diagnosis	5	4	3	2	1	5	4	3	2	1
5. The interpreter does not adequately translate	5	4	3	2	1	5	4	3	2	1
6. The patient presents too many problems	5	4	3	2	1	5	4	3	2	1
7. The patient's history is rambling and disorganized	5	4	3	2	1	5	4	3	2	1
8. The patient does not buy into treatment plan	5	4	3	2	1	5	4	3	2	1
9. The patient provides inconsistent information	5	4	3	2	1	5	4	3	2	1
10. The patient is uninterested in self-care or health maintenance	5	4	3	2	1	5	4	3	2	1
11. Difficulty establishing rapport with the patient	5	4	3	2	1	5	4	3	2	1
12. Difficulty reconciling patient's self-diagnosis with physician's diagnosis	5	4	3	2	1	5	4	3	2	1

	Frequency					Seriousness				
	Always	Most of the time	About half the time	Once in a while	Never	Extremely serious	Very serious	Somewhat serious	Not so serious	None
13. The patient does not want to participate in a partnership with the doctor	5	4	3	2	1	5	4	3	2	1
14. The interpreter is a child or inappropriate	5	4	3	2	1	5	4	3	2	1
15. The patient's cultural beliefs about the illness interfere with the diagnosis and treatment	5	4	3	2	1	5	4	3	2	1
16. The patient does not trust the doctor	5	4	3	2	1	5	4	3	2	1
17. The patient uses culturally based alternative therapies that the doctor is unfamiliar with or disagrees with	5	4	3	2	1	5	4	3	2	1
18. The patient uses a dialect that is difficult to communicate with	5	4	3	2	1	5	4	3	2	1
19. The documentation and ordering of treatment and investigations hinder the flow of the consultation	5	4	3	2	1	5	4	3	2	1
20. The physical set up of the room is not suitable for consultation	5	4	3	2	1	5	4	3	2	1

21. If you believe there are other barriers that hinder your communication with your patients and they are not stated above, please list them in the space below:
