# Supplementary material for: Barriers to effective communication between family physicians and patients in walk-in centre setting in Dubai: a cross-sectional survey
Source: BMC Health Serv Res. 2018 Aug 14;18:637. doi: 10.1186/s12913-018-3457-3 (PMC6092839; doi:10.1186/s12913-018-3457-3)
Supplement: Supplementary file 2 — Table S1. Associations between patients’ gender, nationality, and first language against potential related barriers to communication in the linguistic and cultural factors domain, as well as physicians’ gender (n = 1122). Table S2. Associations between family physicians’ professional level, as well as communication skills training against the time limitation and multiple problem presentation barriers of communication (n = 170). Table S3. Associations between family physicians’ nationality, as well as first language and the inadequate translation barrier to communication (n = 170). Table S4. Associations between patients’ gender and the barriers to communication with family physicians (n = 1122). Table S5. Associations between patients’ age and the barriers to communication with family physicians (n = 1122). (DOC 215 kb) [file 12913_2018_3457_MOESM2_ESM.doc]

**Additional File 2**

**Table S1.** Associations between patients' gender, nationality, and first language against potential related barriers to communication in the linguistic and cultural factors domain, as well as physicians' gender (n=1122).

|  |  |  | **Barriers:** | **Difficulty understanding language** | | | | **Lack of cultural understanding** | | | | **Physician's gender** | | | | **Physician's culture** | | | |
| --- | --- | --- | --- | --- | --- | --- | --- | --- | --- | --- | --- | --- | --- | --- | --- | --- | --- | --- | --- |
| **Category** | **Subcategory** | **Total** | | **H-A** | | **O-N** | | **H-A** | | **O-N** | | **H-A** | | **O-N** | | **H-A** | | **O-N** | |
|  |  | **Frequency** | **%** | **n** | **%** | **n** | **%** | **n** | **%** | **n** | **%** | **n** | **%** | **n** | **%** | **n** | **%** | **n** | **%** |
| **Gender** | Male | 356 | 31.7% | 39 | 11.0% | 317 | 89.0% | 36 | 10.1% | 320 | 89.9% | 56 | 15.7% | 300 | 84.3% | 58 | 16.3% | 298 | 83.7% |
|  | Female | 766 | 68.3% | 96 | 12.5% | 670 | 87.5% | 94 | 12.3% | 672 | 87.7% | 104 | 13.6% | 662 | 86.4% | 119 | 15.5% | 647 | 84.5% |
|  |  |  | ***p- value*** |  |  |  | ***0.50*** |  |  |  | ***0.32*** |  |  |  | ***0.36*** |  |  |  | ***0.80*** |
|  |  |  |  |  |  |  |  |  |  |  |  |  |  |  |  |  |  |  |  |
| **Nationality** | UAE | 866 | 77.2% | 106 | 12.2% | 760 | 87.8% | 109 | 12.6% | 757 | 87.4% | 126 | 14.6% | 740 | 85.5% | 139 | 16.1% | 727 | 84.0% |
|  | Other | 256 | 22.8% | 29 | 11.3% | 227 | 88.7% | 21 | 8.2% | 235 | 91.8% | 34 | 13.3% | 222 | 86.7% | 38 | 14.8% | 218 | 85.2% |
|  |  |  | ***p- value*** |  |  |  | ***0.74*** |  |  |  | ***0.06*** |  |  |  | ***0.68*** |  |  |  | ***0.70*** |
|  |  |  |  |  |  |  |  |  |  |  |  |  |  |  |  |  |  |  |  |
| **First language** | Arabic | 964 | 85.9% | 119 | 12.3% | 845 | 87.7% | 115 | 11.9% | 849 | 88.1% | 143 | 14.8% | 821 | 85.2% | 154 | 16.0% | 810 | 84.0% |
|  | Other | 158 | 14.1% | 16 | 10.1% | 142 | 89.9% | 15 | 9.5% | 143 | 90.5% | 17 | 10.8% | 141 | 89.2% | 23 | 14.6% | 135 | 85.4% |
|  |  |  | ***p- value*** |  |  |  | ***0.51*** |  |  |  | ***0.42*** |  |  |  | ***0.22*** |  |  |  | ***0.73*** |

*Abbreviations:- H-A: half of the time-always; O-N: once in a while-never.*

**Table S2.** Associations between family physicians’ professional level, as well as communication skills training against the time limitation and multiple problem presentation barriers of communication (n=170).

|  |  |  |  | **Barriers:** | **Time limitation** | | | | **Multiple problems presentation** | | | |
| --- | --- | --- | --- | --- | --- | --- | --- | --- | --- | --- | --- | --- |
| **Category** | **Subcategory** |  | **Total** |  | **Very Low-Low** | | **Moderate-Very High** | | **Very Low-Low** | | **Moderate-Very High** | |
|  |  |  | **Frequency** | **%** | **n** | **%** | **n** | **%** | **n** | **%** | **n** | **%** |
| **Professional level** | Senior resident |  | 25 | 14.7% | 11 | 44.0% | 14 | 56.0% | 10 | 40.0% | 15 | 60.0% |
|  | Specialist registrar |  | 72 | 42.4% | 33 | 45.8% | 39 | 54.2% | 37 | 51.4% | 35 | 48.6% |
|  | Senior specialist registrar |  | 60 | 35.3% | 32 | 53.3% | 28 | 46.7% | 31 | 51.7% | 29 | 48.3% |
|  | Consultant |  | 13 | 7.7% | 8 | 61.5% | 5 | 38.5% | 5 | 38.5% | 8 | 61.5% |
|  |  |  |  | ***χ^2^ [3]*** |  |  |  | ***1.80*** |  |  |  | ***1.72*** |
|  |  |  |  | ***p- value*** |  |  |  | ***0.18*** |  |  |  | ***0.19*** |
|  |  |  |  |  |  |  |  |  |  |  |  |  |
| **Communication skills training** | Medical school | Yes | 105 | 61.8% | 49 | 46.7% | 56 | 53.3% | 48 | 45.7% | 57 | 54.3% |
|  |  | No | 65 | 38.2% | 35 | 53.9% | 30 | 46.2% | 35 | 53.9% | 30 | 46.2% |
|  |  |  |  | ***p- value*** |  |  |  | ***0.43*** |  |  |  | ***0.35*** |
|  |  |  |  |  |  |  |  |  |  |  |  |  |
|  | Postgraduate/ residency | Yes | 151 | 88.8% | 79 | 52.3% | 72 | 47.7% | 74 | 49.0% | 77 | 51.0% |
|  |  | No | 19 | 11.2% | 5 | 26.3% | 14 | 73.7% | 9 | 47.4% | 10 | 52.6% |
|  |  |  |  | ***p- value*** |  |  |  | ***0.05*** |  |  |  | ***1.0*** |
|  |  |  |  |  |  |  |  |  |  |  |  |  |
|  | Within last year | Yes | 61 | 35.9% | 34 | 55.7% | 27 | 44.3% | 28 | 45.9% | 33 | 54.1% |
|  |  | No | 109 | 64.1% | 50 | 45.9% | 59 | 54.1% | 55 | 50.5% | 54 | 49.5% |
|  |  |  |  | ***p- value*** |  |  |  | ***0.26*** |  |  |  | ***0.63*** |

**Table S3.** Associations between family physicians' nationality, as well as first language and the inadequate translation barrier to communication (n=170).

|  |  |  | **Barriers:** | **Inadequate translation by interpreter** | | | |
| --- | --- | --- | --- | --- | --- | --- | --- |
| **Category** | **Subcategory** | **Total** |  | **Very Low-Low** | | **Moderate-Very High** | |
|  |  | **Frequency** | **%** | **n** | **%** | **n** | **%** |
| **Nationality** | United Arab Emirates | 75 | 44.1% | 58 | 77.3% | 17 | 22.7% |
|  | Other | 95 | 55.9% | 86 | 90.5% | 9 | 9.5% |
|  |  |  | ***p- value*** |  |  |  | ***0.03*** |
|  |  |  |  |  |  |  |  |
| **First language** | Arabic | 138 | 81.2% | 113 | 81.9% | 25 | 18.1% |
|  | Other | 32 | 18.8% | 31 | 96.9% | 1 | 3.1% |
|  |  |  | ***p- value*** |  |  |  | ***0.03*** |

**Table S4.** Associations between patients’ gender and the barriers to communication with family physicians (n=1122).

|  |  | **Gender** | ***Male*** | ***Female*** |  |  |  |  |
| --- | --- | --- | --- | --- | --- | --- | --- | --- |
|  | **Total** | **Count** | 356 | 766 |  |  |  |  |
|  |  | **%** | 31.7% | 68.3% |  |  |  |  |
| **Barriers** |  |  | **Half of the time-Always** | | **Once in a while-Never** | |  |  |
|  |  |  | ***Male %*** | ***Female %*** | ***Male %*** | ***Female %*** | ***χ^2^ [1]*** | ***p- value*** |
| Time limitation | |  | **19.4%** | **25.3%** | 80.6% | 74.7% | 4.46 | **<0.05** |
|  |  |  |  |  |  |  |  |  |
| Large amount of information | | | **13.8%** | **18.4%** | 86.2% | 81.6% | 3.40 | **0.07** |
|  |  |  |  |  |  |  |  |  |
| Medical jargon use | |  | **9.0%** | **13.8%** | 91.0% | 86.2% | 4.86 | **<0.05** |
|  |  |  |  |  |  |  |  |  |
| Difficulty understanding language | | | **11.0%** | **12.5%** | 89.0% | 87.5% | 0.43 | **0.51** |
|  |  |  |  |  |  |  |  |  |
| Preoccupation with computer | | | **13.2%** | **12.1%** | 86.8% | 87.9% | 0.16 | **0.69** |
|  |  |  |  |  |  |  |  |  |
| Lack of interest in issues raised | | | **15.2%** | **15.3%** | 84.8% | 84.7% | 0.00 | **0.97** |
|  |  |  |  |  |  |  |  |  |
| Rapid talking | |  | **12.6%** | **14.5%** | 87.4% | 85.5% | 0.55 | **0.46** |
|  |  |  |  |  |  |  |  |  |
| Denying chance to talk | | | **10.4%** | **15.1%** | 89.6% | 84.9% | 4.26 | **<0.05** |
|  |  |  |  |  |  |  |  |  |
| Not checking understanding | | | **12.9%** | **17.5%** | 87.1% | 82.5% | 3.44 | **0.06** |
|  |  |  |  |  |  |  |  |  |
| Not being empathic | |  | **12.4%** | **16.3%** | 87.6% | 83.7% | 2.68 | **0.10** |
|  |  |  |  |  |  |  |  |  |
| Not addressing all issues | | | **11.8%** | **16.7%** | 88.2% | 83.3% | 4.19 | **<0.05** |
|  |  |  |  |  |  |  |  |  |
| Inability to understand the problem | | | **11.2%** | **16.4%** | 88.8% | 83.6% | 4.83 | **<0.05** |
|  |  |  |  |  |  |  |  |  |
| Unsatisfactory manners | | | **8.7%** | **12.1%** | 91.3% | 87.9% | 2.58 | **0.11** |
|  |  |  |  |  |  |  |  |  |
| Pressurising for decisions | | | **9.8%** | **11.9%** | 90.2% | 88.1% | 0.83 | **0.36** |
|  |  |  |  |  |  |  |  |  |
| Lack of aid tools | |  | **13.2%** | **18.7%** | 86.8% | 81.3% | 4.78 | **<0.05** |
|  |  |  |  |  |  |  |  |  |
| Lack of cultural understanding | | | **10.1%** | **12.3%** | 89.9% | 87.7% | 0.91 | **0.34** |
|  |  |  |  |  |  |  |  |  |
| Physician's gender | |  | **15.7%** | **13.6%** | 84.3% | 86.4% | 0.75 | **0.39** |
|  |  |  |  |  |  |  |  |  |
| Physician's culture | |  | **16.3%** | **15.5%** | 83.7% | 84.5% | 0.06 | **0.81** |

**Table S5.** Associations between patients’ age and the barriers to communication with family physicians (n=1122).

|  |  | **Age (in years)** | **18-30** | **31-40** | **41-50** | **>50** |  |  |  |  |  |  |
| --- | --- | --- | --- | --- | --- | --- | --- | --- | --- | --- | --- | --- |
|  | **Total** | **Count** | 461 | 335 | 216 | 110 |  |  |  |  |  |  |
|  |  | **%** | 41.1% | 29.9% | 19.3% | 9.8% |  |  |  |  |  |  |
| **Barriers** | |  | **Half of the time-Always** | | | | **Once in a while-Always** | | | |  |  |
|  |  |  | **18-30** (%) | **31-40** (%) | **41-50** (%) | **>50** (%) | **18-30** (%) | **31-40** (%) | **41-50** (%) | **>50** (%) | ***χ^2^ [3]*** | **p- value** |
| Time limitation | | | **28.2%** | **22.7%** | **18.1%** | **16.4%** | 71.8% | 77.3% | 81.9% | 83.6% | 12.48 | **<0.05** |
|  |  |  |  |  |  |  |  |  |  |  |  |  |
| Large amount of information | | | **21.5%** | **16.7%** | **10.6%** | **10.9%** | 78.5% | 83.3% | 89.4% | 89.1% | 15.68 | **<0.05** |
|  |  |  |  |  |  |  |  |  |  |  |  |  |
| Medical jargon use | | | **16.5%** | **10.1%** | **9.3%** | **7.3%** | 83.5% | 89.9% | 90.7% | 92.7% | 13.35 | **<0.05** |
|  |  |  |  |  |  |  |  |  |  |  |  |  |
| Difficulty understanding language | | | **16.9%** | **9.6%** | **8.3%** | **6.4%** | 83.1% | 90.4% | 91.7% | 93.6% | 18.48 | **<0.05** |
|  |  |  |  |  |  |  |  |  |  |  |  |  |
| Preoccupation with computer | | | **17.4%** | **9.3%** | **9.7%** | **7.3%** | 82.6% | 90.7% | 90.3% | 92.7% | 17.46 | **<0.05** |
|  |  |  |  |  |  |  |  |  |  |  |  |  |
| Lack of interest in issues raised | | | **19.3%** | **14.9%** | **10.6%** | **8.2%** | 80.7% | 85.1% | 89.4% | 91.8% | 13.69 | **<0.05** |
|  |  |  |  |  |  |  |  |  |  |  |  |  |
| Rapid talking | | | **19.1%** | **12.2%** | **9.3%** | **6.4%** | 80.9% | 87.8% | 90.7% | 93.6% | 20.25 | **<0.05** |
|  |  |  |  |  |  |  |  |  |  |  |  |  |
| Denying chance to talk | | | **17.4%** | **14.3%** | **8.8%** | **5.5%** | 82.6% | 85.7% | 91.2% | 94.5% | 16.09 | **<0.05** |
|  |  |  |  |  |  |  |  |  |  |  |  |  |
| Not checking understanding | | | **20.2%** | **16.4%** | **10.6%** | **8.2%** | 79.8% | 83.6% | 89.4% | 91.8% | 15.59 | **<0.05** |
|  |  |  |  |  |  |  |  |  |  |  |  |  |
| Not being empathic | | | **18.7%** | **15.8%** | **9.3%** | **9.1%** | 81.3% | 84.2% | 90.7% | 90.9% | 13.55 | **<0.05** |
|  |  |  |  |  |  |  |  |  |  |  |  |  |
| Not addressing all issues | | | **17.6%** | **16.4%** | **10.2%** | **10.9%** | 82.4% | 83.6% | 89.8% | 89.1% | 8.20 | **<0.05** |
|  |  |  |  |  |  |  |  |  |  |  |  |  |
| Inability to understand the problem | | | **19.3%** | **14.3%** | **9.3%** | **8.2%** | 80.7% | 85.7% | 90.7% | 91.8% | 16.57 | **<0.05** |
|  |  |  |  |  |  |  |  |  |  |  |  |  |
| Unsatisfactory manners | | | **13.9%** | **11.3%** | **7.9%** | **4.5%** | 86.1% | 88.7% | 92.1% | 95.5% | 10.75 | **<0.05** |
|  |  |  |  |  |  |  |  |  |  |  |  |  |
| Pressurising for decisions | | | **13.4%** | **10.7%** | **9.3%** | **7.3%** | 86.6% | 89.3% | 90.7% | 92.7% | 4.93 | **<0.05** |
|  |  |  |  |  |  |  |  |  |  |  |  |  |
| Lack of aid tools | |  | **18.2%** | **21.5%** | **9.3%** | **12.7%** | 81.8% | 78.5% | 90.7% | 87.3% | 15.92 | **<0.05** |
|  |  |  |  |  |  |  |  |  |  |  |  |  |
| Lack of cultural understanding | | | **14.8%** | **10.7%** | **8.8%** | **6.4%** | 85.2% | 89.3% | 91.2% | 93.6% | 9.31 | **<0.05** |
|  |  |  |  |  |  |  |  |  |  |  |  |  |
| Physician's gender | | | **16.1%** | **12.8%** | **13.0%** | **13.6%** | 83.9% | 87.2% | 87.0% | 86.4% | 2.10 | **0.15** |
|  |  |  |  |  |  |  |  |  |  |  |  |  |
| Physician's culture | | | **19.3%** | **15.8%** | **11.1%** | **10.0%** | 80.7% | 84.2% | 88.9% | 90.0% | 10.62 | **<0.05** |
